# [^125^I]IPC-Lecanemab: Synthesis and Evaluation of Aβ-Plaque-Binding Antibody and Comparison with Small-Molecule [^18^F]Flotaza and [^125^I]IBETA in Postmortem Human Alzheimer’s Disease

**DOI:** 10.3390/neurolint16020031

**Published:** 2024-04-08

**Authors:** Christopher Liang, Cayz G. Paclibar, Noresa L. Gonzaga, Stephanie A. Sison, Harman S. Bath, Agnes P. Biju, Jogeshwar Mukherjee

**Affiliations:** Preclinical Imaging, Department of Radiological Sciences, University of California-Irvine, Irvine, CA 92697, USA; liangc@uci.edu (C.L.); cgpaclib@uci.edu (C.G.P.); nlgonzag@uci.edu (N.L.G.); sisonsa@uci.edu (S.A.S.); bathhs@uci.edu (H.S.B.); apbiju@uci.edu (A.P.B.)

**Keywords:** Lecanemab, Aducanumab, iodine-125, human postmortem AD, PET, autoradiography

## Abstract

Therapeutic antibodies for reducing Aβ plaque load in Alzheimer’s disease (AD) is currently making rapid progress. The diagnostic imaging of Aβ plaque load in AD has been underway and is now used in clinical studies. Here, we report our preliminary findings on imaging a therapeutic antibody, Lecanemab, in a postmortem AD brain anterior cingulate. [^125^I]5-iodo-3-pyridinecarboxamido-Lecanemab ([^125^I]IPC-Lecanemab) was prepared by coupling *N*-succinimidyl-5-([^125^I]iodo)-3-pyridinecarboxylate with Lecanemab in modest yields. The distinct binding of [^125^I]IPC-Lecanemab to Aβ-rich regions in postmortem human AD brains was higher in grey matter (GM) containing Aβ plaques compared to white matter (WM) (GM/WM was 1.6). Anti-Aβ immunostaining was correlated with [^125^I]IPC-Lecanemab regional binding in the postmortem AD human brains. [^125^I]IPC-Lecanemab binding was consistent with the binding of Aβ small molecules, [^18^F]flotaza and [^125^I]IBETA, in the same subjects. [^18^F]Flotaza and [^125^I]IBETA, however, exhibited significantly higher GM/WM ratios (>20) compared to [^125^I]IPC-Lecanemab. Our results suggest that radiolabeled [^125^I]IPC-Lecanemab retains the ability to bind to Aβ in human AD and may therefore be useful as a PET imaging radiotracer when labeled as [^124^I]IPC-Lecanemab. The ability to directly visualize in vivo a promising therapeutic antibody for AD may be useful in treatment planning and dosing and could be complimentary to small-molecule diagnostic imaging to assess outcomes of therapeutic interventions.

## 1. Introduction

**Figure 1:**
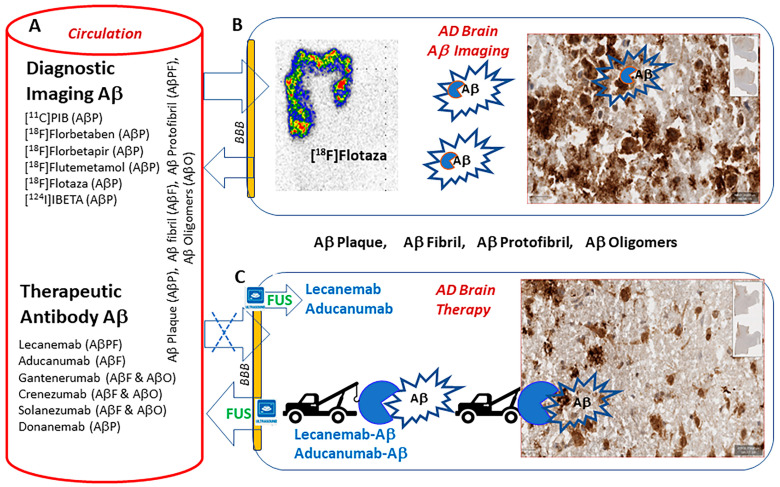
Diagnostic and therapeutic strategies for Aβ in AD subjects. (**A**) Intravenously administered PET imaging agents radiolabeled with carbon-11, fluorine-18 and iodine-124 and therapeutic antibodies in circulation may have the ability to bind to the various Aβ components (Aβ plaques (AβP), Aβ fibrils (AβF), Aβ protofibrils (AβPF), Aβ oligomers (AβO)). (**B**) Small radiolabeled diagnostic molecules such as [^18^F]flotaza freely cross the blood–brain barrier (BBB) and bind to Aβ plaques (high binding in red-yellow) and provide quantitative analysis of Aβ plaque load in the AD brain. (**C**) Intravenously administered therapeutic antibodies do not cross the BBB but bind to various Aβ components (AβP, AβF, AβPF, AβO) outside the brain. Recent work in human subjects has used focused ultrasound (FUS) to make the BBB permeable, enabling Lecanemab and Aducanumab to permeate the brain. This allowed binding of the antibodies to Aβ components and towing them out of the brain while the BBB was open using FUS.

Until recently, approved Food and Drug Administration (FDA) drugs for Alzheimer’s disease (AD) treatment only included acetylcholinesterase inhibitors [1]. Reductions in the accumulation of Aβ amyloid plaques continue to be investigated as potential therapeutic approaches for AD [2]. Using antibodies such as Lecanemab, recent efforts to reduce Aβ plaque load in early AD patients appears promising and has recently gained FDA approval [3]. The findings with Lecanemab (unlike previously approved antibodies) suggest clinical benefit [4], but many questions still remain, including the possibility of prolonged intravenous antibody infusions [5]. Small-molecule treatments for reducing Aβ plaque load may potentially be more favorable for patient comfort, compliance, safety and cost. However, approaches such as inhibitors of β- and γ-secretases have not shown promise in altering the course of Aβ plaque accumulation [6]. We have previously proposed the development of multi-targeting agents for Aβ plaque removal in AD, which would comprise an Aβ-plaque-targeting agent that is tethered to a second target agent, P-glycoprotein (Pgp), to assist in the removal of the plaque components from the brain and the surrounding vasculature across the blood–brain barrier (BBB), as shown in Figure 1 [7]. Decreased Pgp function in AD adds to the Aβ burden through decreased Aβ efflux (oligomers, fibrils and plaques) across the BBB [8]. This insufficient clearance of macromolecules such as Aβ results in the formation of Aβ fibrils and plaques in the brain. The downregulation of Pgp has been reported in AD, and the upregulation of Pgp using substrates for Pgp was found to increase the clearance of Aβ from the brain [9,10].

Diagnostic imaging, positron emission tomography (PET) and single-photon emission computed tomography (SPECT) based on small molecules (Figure 1) that easily cross the BBB have played a major role in understanding the development of Aβ plaques [11]. Over the last decade, the diagnostic imaging of Aβ plaques and related precursor components in the various stages of AD has made tremendous progress [12]. Information on the progression of AD at various Braak stages and the involvement of different brain regions has provided a wealth of information on relating the stages of the disease versus Aβ load [13]. The successful development and use of diagnostic imaging agents, as outlined in Figure 1, have enabled the monitoring of Aβ load. Efforts still continue in the development of small molecules in attempts to further enhance the target-to-nontarget contrast ratio in the brain regions that may increase the sensitivity to detect changes in Aβ [14,15,16]. PET imaging agents for the visualization of Aβ protofibrils and Aβ oligomers have yet to be demonstrated.

In contrast to diagnostic imaging, the therapeutic success of removing Aβ aggregates and other Aβ components has remained more challenging. Figure 1 shows several antibodies that target Aβ [17]. Antibodies such as Lecanemab, Aducanumab and Gantenerumab have nanomolar affinities for small and large Aβ protofibrils, Aβ fibrils and Aβ plaques, while all of them have weak micromolar affinities for the Aβ monomer. Lecanemab, which a has preferential affinity for Aβ protofibrils, has shown significant efficacy recently [3,4]. The inability of the therapeutic antibodies to efficiently permeate into the brain has been a significant limiting factor. Radiolabeled Lecanemab may be useful for in vivo imaging by PET and may allow for biodistribution studies. Such studies may allow for assessments of dosing and treatment responses in patients being treated with Lecanemab. 

Focused ultrasound (FUS) is now being used to overcome the problems of the transport of the antibodies across the BBB. It has been used to increase BBB permeability temporarily in order to increase the efficacy of Aβ plaque removal [18,19]. Therapeutic FUS using intravenously injected microbubbles and Aducanumab in transgenic AD mice reduced Aβ plaques and improved spatial memory [20]. Focused ultrasound has now been applied in a limited number of AD patients undergoing Aducanumab therapy to assist in Aβ removal from the brain [21]. Lecanemab and Aducanumab are both of current interest due to their promising efficacy in early-stage Alzheimer’s disease [22], despite a few reports of amyloid-related imaging abnormalities (ARIA) [23]. Radiolabeled Lecanemab may be used to ascertain dosing, brain uptake and pharmacokinetics of the therapeutic doses. Radiolabeled Lecanemab may also be useful to assess efficacy of FUS when administering therapeutic doses of Lecanemab and Aducanumab. 

Thus, our eventual goal is to develop an in vivo imaging agent based on Lecanemab that can be labeled using iodine-123 (for SPECT) and iodine-124 (for PET) imaging studies. As a first step, our objective in this work was to demonstrate the radiolabeling of Lecanemab with iodine-125 and assess the binding of iodine-125-labeled Lecanemab to Aβ in human postmortem AD brains. Iodine-125-labeled Lecanemab was also compared with the small-molecule Aβ-imaging agents [^18^F]flotaza [14] and [^125^I]IBETA [15], which have been shown to bind to postmortem human brain Aβ plaques. Iodine-125 was incorporated in an aromatic ring using a previously reported *N*-succinimidyl-5-(tributylstannyl)-3-pyridinecarboxylate (SPC) procedure [24] and then coupled to Lecanemab. We report here the preparation and evaluation of iodine-125-radiolabeled [^125^I]5-iodo-3-pyridinecarboxamido-Lecanemab ([^125^I]IPC-Lecanemab). The binding of [^125^I]IPC-Lecanemab was compared with the small-molecule Aβ-PET-imaging agents [^18^F]flotaza [14] and [^124/125^I]IBETA [15] to assess its binding to Aβ in the same postmortem human AD brain anterior cingulate brain slices. 

## 2. Materials and Methods

### 2.1. General Methods

Lecanemab-biosimilar anti-amyloid β protofibril mAb (code PX-TA1746; lot 100822-A01) was purchased from ProteoGenix, France. This monoclonal antibody is of the isotype IgG1 with the origin species of Homo sapiens and the expression system XtenCHO and goes by the synonyms of Lecanemab or BAN2401 (CAS 1260393-98-3). *N*-succinimidyl-5-(tributylstannyl)-3-pyridinecarboxylate was purchased from AABlocks, Inc., San Diego, CA, USA. All chemicals and solvents were purchased from Aldrich Chemical and Fisher Scientific. Deionized water was acquired from a Millipore Milli-Q Water Purification System. Iodine-125 sodium iodide (specific activity = 643 MBq/μg) in 0.01N NaOH was purchased from American Radiolabeled Chemicals, Inc., St. Louis, MO, USA. All radioiodinations were carried out in a CBS radioiodination hood equipped with a charcoal filter and exhaust fan. This radioiodination hood was placed inside a fume hood that was equipped with an extensive filtration system (prefilter, HEPA filter and charcoal filter). Iodine-125 radioactivity was counted in a Capintec CRC-15R dose calibrator, while low-level counting was carried out in a Capintec Caprac-R well-counter. Analytical thin-layer chromatography (TLC) was used to monitor the reactions (Baker-flex, Phillipsburg, NJ, USA). The mass spectrometry analysis used an ESI LC-ToF Micromass LCT Premier and Xevo G2-XS ToF MS (Waters Co., Milford, MA, USA). RadioTLCs were scanned on an AR-2000 imaging scanner (Eckart & Ziegler, Berlin, Germany). 

### 2.2. Radiosynthesis of [^125^I]IPC-Lecanemab

**Figure 2:**
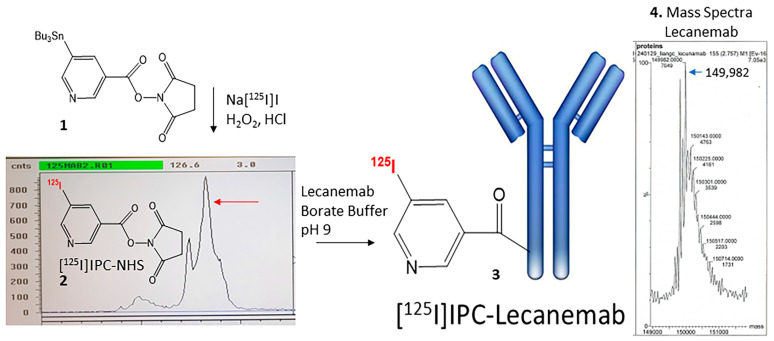
Radiolabeling of Lecanemab: *N*-succinimidyl-5-(tri-butylstannyl)-3-pyridinecarboxylate (**1**) was reacted with [^125^I]sodium iodide under electrophilic conditions to provide *N*-succinimidyl-5-[^125^I]iodo-3-pyridinecarboxylate (**2**). RadioTLC shows [^125^I]PIC-NHS in >80% purity (red arrow). Lecanmab (ProteoGenix from code PX-TA1746; lot 100822-A01), as confirmed by protein mass spectrometry (mass 149,982), was radiolabeled using [^125^I]IPC-NHS (**2**) to provide [^125^I]5-iodo-3-pyridinecarboxamido-Lecanemab ([^125^I]IPC-Lecanemab (**3**)).

Sodium iodide, [^125^I]NaI (ARC Inc.), was used to radiolabel the bifunctional linker *N*-succinimidyl-5-(tributylstannyl)-3-pyridinecarboxylate (SPC) (**1**) by electrophilic substitution of the tributyltin derivative using reported radioiodination methods [15]. The radiosynthesis of *N*-succinimidyl-5-([^125^I]iodo)-3-pyridinecarboxylate ([^125^I]IPC-NHS) (**2**) was carried out by adding 0.1 mL H_2_O_2_ (3%) to a mixture of 0.1 mL of tributyltin derivative (1 mg/1 mL of ethanol), 6 MBq [^125^I]NaI and 0.1 mL of 1N HCl in a sealed vial. The reaction was allowed to proceed at room temperature for 30 min before it was terminated by the addition of sodium bisulfite. Two rounds of extraction were carried out using dichloromethane. The extract was then dried using anhydrous MgSO_4_. The residue, 3 MBq of *N*-succinimidyl-5-([^125^I]iodo)-3-pyridinecarboxylate, was obtained and verified by radioTLC (Figure 2). This was taken in ethanol (10 μL) in an Eppendorf tube and was used to react with Lecanemab. Lecanemab-biosimilar anti-amyloid β protofibril mAb (50 μg; 33 μM) was taken in borate buffer (50 μL, 100 mM, pH 9) (final concentration of Lecanemab in the reaction mixture was 5.5 μM) in an Eppendorf vial. This mixture was added to the Eppendorf vial containing 1.4 MBq of [^125^I]IPC-NHS and allowed to react for one hour at room temperature. All the [^125^I]IPC-NHS (**2**) was consumed, and this was confirmed by radioTLC. The product, [^125^I]IPC-Lecanemab (**3**), was diluted in PBS buffer, pH 7.4, and used for in vitro experiments.

### 2.3. Human Tissue

Human postmortem brain tissue samples for in vitro experiments were obtained from the brain tissue repository of Banner Sun Health Research Institute (BHRI), Sun City, AZ, [25]. Well-characterized AD brain and cognitively normal (CN) brain tissue samples of the anterior cingulate cortex were used for this study. The subjects’ Aβ plaque totals included neuritic, cored and diffuse in the frontal, temporal, parietal, hippocampal and entorhinal cortex. Semi-quantitative scores of none, sparse, moderate and frequent were converted to numerical values 0–3 for each region and summed to the provide Aβ plaque total for each AD and CN subject. Similarly, the tangle totals included the neurofibrillary tangle density in the frontal, temporal and parietal lobes, the hippocampal CA1 region and the entorhinal cortical regions. Numerical values 0–3 for each region were summed to provide the tangle total for each AD and CN subject. Based on these values, the Braak scores of the CN subjects were I-II, and those of the AD subjects were V–VI. Further details of these subjects have been previously reported [26].

Brain slices that were 10 µm thick were obtained from chunks of frozen tissue on a Leica 1850 cryotome cooled to −20 °C. Iodine-125 autoradiographic studies were carried out by exposing the tissue samples on storage phosphor screens (Perkin Elmer Multisensitive, Medium MS and tritium-sensitive phosphor screens). The apposed phosphor screens were read and analyzed by the OptiQuant acquisition and analysis program of the Cyclone Storage Phosphor System to measure the binding of [^125^I]IPC-Lecanemab as digital light units (DLU)/mm^2^ (Packard Instruments Co., Boston, MA, USA). Adjacent slices were used for immunostaining with anti-Aβ. All postmortem human brain studies were approved by the Institutional Biosafety Committee of University of California, Irvine.

### 2.4. Postmortem Human Brain Autoradiography

#### 2.4.1. [^125^I]IPC-Lecanemab

Human AD (*n* = 6) and cognitively normal (CN, *n* = 6) postmortem brain tissues were obtained from BHRI, Sun City, AZ, USA. Brain slices were sectioned (10 μm thickness) on a Leica 1850 Cryostat (Leica Biosystems, Deerfield, IL, USA) and collected on Fisher slides. The slides contained from one to three brain sections each and were placed in separate glass chambers (six slides per chamber) and were preincubated in PBS buffer containing 0.2% bovine serum albumin (BSA), pH 7.4, at 25 °C for 15 min. The preincubation buffer was discarded. The brain slices were then treated with [^125^I]IPC-Lecanemab (final concentration of 1.8 nM) in PBS buffer containing 0.2% BSA, pH 7.4, at 25 °C (60 mL, 1.5 kBq/mL). The chambers were incubated at 25 °C for 2 h. The slices were then washed with cold 0.2% BSA PBS buffer, pH 7.4, three times for 10 min each time, followed by an ice-cold water rinse. The brain sections were air-dried and apposed on a phosphor film for two weeks (Multisensitive Medium MS, PerkinElmer, Waltham, MA, USA). The apposed phosphor screens were read and analyzed by OptiQuant acquisition and the Cyclone Storage Phosphor System (Packard Instruments Co., Boston, MA, USA). ROIs were drawn on the slices, and the extent of binding of [^125^I]IPC-Lecanemab was measured in DLU/mm^2^. Nonspecific binding was determined by using the white matter region, the corpus callosum, as a reference region.

#### 2.4.2. [^18^F]Flotaza

In vitro imaging studies using [^18^F]flotaza on this cohort of AD and CN subjects have been previously reported [14].

#### 2.4.3. [^125^I]IBETA and [^124^I]IBETA

Procedures for [^125^I]IBETA and [^124^I]IBETA have been reported previously on this cohort of AD and CN of subjects [15].

### 2.5. Immunohistochemistry

Anti-Aβ immunostaining of all brain sections was carried out by the pathology services of the University of California-Irvine using the Ventana BenchMark Ultra protocols. Immunostained sections were scanned using the Ventana Roche instrumentation, and the images were analyzed using the QuPath software version 0.5.1 [16].

### 2.6. Image Analysis

The group differences between the AD and CN subjects were determined using Microsoft Excel 16 and GraphPad Prism 9. The statistical power was determined with Student’s *t*-test, and *p* values of <0.05 were considered to indicate statistical significance.

## 3. Results

We developed efficient radioiodination procedures for [^125^I]-, [^124^I]- and [^123^I-labeled compounds for various small molecules, including Aβ plaque and Tau imaging agents [27,28,29]. These procedures involved the electrophilic substitution of an aromatic tributylstannyl derivative of the small molecule by radioiodine. In order to radiolabel proteins, either direct radiolabeling with radioiodine has been pursued or a more stable bi-functional linker, SPC, has been used [24]. We chose to use the SPC approach in order to radiolabel Lecanemab so that its binding properties to Aβ would be preserved and that the radioiodine would be more stable. Hydrogen peroxide was used as an oxidant in the radiolabeling with [^125^I]NaI. At room temperature, the radiolabeling with [^125^I]NaI proceeded for 30 min before being terminated. RadioTLC was used to monitor the reaction progress, and a purity of >85% was shown by the radioTLC (Figure 2). Some hydrolysis of the succinimide ester resulted in a small left shoulder in the reaction mixture. The radiochemical yield of the radioiodinated product, *N*-succinimidyl-5-([^125^I]iodo)-3-pyridinecarboxylate ([^125^I]IPC-NHS), was approximately 40–50%.

Lecanemab (mass of 149,982, Figure 2(4) mass spectrum, consistent with reported 150 kDa) was reacted under mild conditions in borate buffer with [^125^I]IPC-NHS. The reaction of [^125^I]IPC-NHS into Lecanemab to provide [^125^I]IPC-Lecanemab resulted in a good yield. RadioTLC determined that all the [^125^I]IPC-NHS was consumed in the efficient coupling of the bifunctional chelator. Previous reports on the radiolabeling of murine mAb158- and mAb158-transferrin receptor conjugates used chloramine-T for the direct electrophilic incorporation of iodine-125 and iodine-124 [30,31].

**Figure 3:**
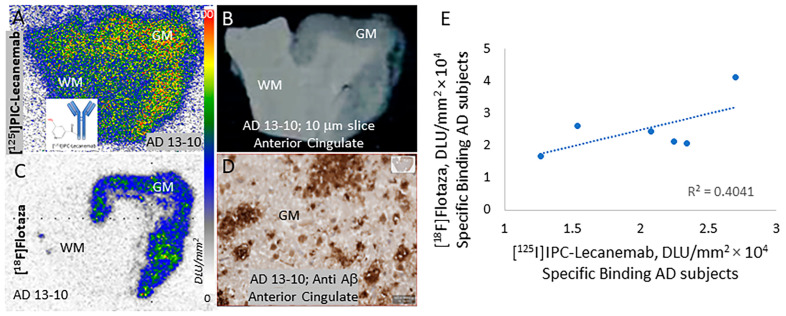
[^125^I]IPC-Lecanemab and [^18^F]flotaza binding in human postmortem AD. (**A**) [^125^I]IPC-Lecanemab binding to anterior cingulate (AC) of AD 13-10 (red-yellow shows higher binding) with no binding to corpus callosum (CC) (blue shows nonspecific binding). (**B**) Corresponding scanned brain slice of AD 13-10 showing GM and WM. (**C**) [^18^F]Flotaza binding to anterior cingulate (AC) of AD 13-10 (yellow-green-blue shows higher binding) with no binding to corpus callosum (CC) (grey-white shows nonspecific binding). (**D**) Anti-Aβ IHC showing pixel thresholder in inset of AD 13-10. (**E**) Correlation plot of [^18^F]flotaza and [^125^I]IPC-Lecanemab binding in six AD subjects.

The binding of [^125^I]IPC-Lecanemab to Aβ-rich regions was seen in the anterior cingulate regions of the six AD subjects. The average ratio of [^125^I]IPC-Lecanemab binding in the grey matter (anterior cingulate) to the white matter (corpus callosum) was 1.6. The radiolabeled antibodies, due to their size, exhibited a significant amount of nonspecific binding, as can be seen in the white matter (Figure 3A). Lecanemab had a high affinity for Aβ protofibrils (IC_50_ = 0.8 nM) and Aβ fibrils (IC_50_ = 1.8 nM) but a weaker affinity for Aβ plaques [3]. The binding of [^125^I]IPC-Lecanemab in the grey matter may be reflective of Aβ protofibrils, Aβ fibrils and Aβ plaques. However, since anti-Aβ immunostains of the AD subjects revealed the presence of extensive amounts of diffuse and neuritic Aβ plaques (Figure 3B,D), the low GM/WM ratio may be reflective of the weak affinity of [^125^I]IPC-Lecanemab for Aβ plaques. Figure 3C shows the binding of [^18^F]flotaza in the same AD subject. The high levels of [^18^F]flotaza binding were consistent with immunostaining in adjacent sections. Since the white matter binding was very small across all the AD subjects, the ratios of [^18^F]flotaza between the anterior cingulate and corpus callosum were found to be >70 in all the subjects. The plot of the specific binding of [^18^F]flotaza versus [^125^I]IPC-Lecanemab in the six AD subjects is shown in Figure 3E. The poor correlation may be a reflective of both the extent of the specific binding and the differences in the Aβ targets for [^18^F]flotaza and [^125^I]IPC-Lecanemab.

**Figure 4:**
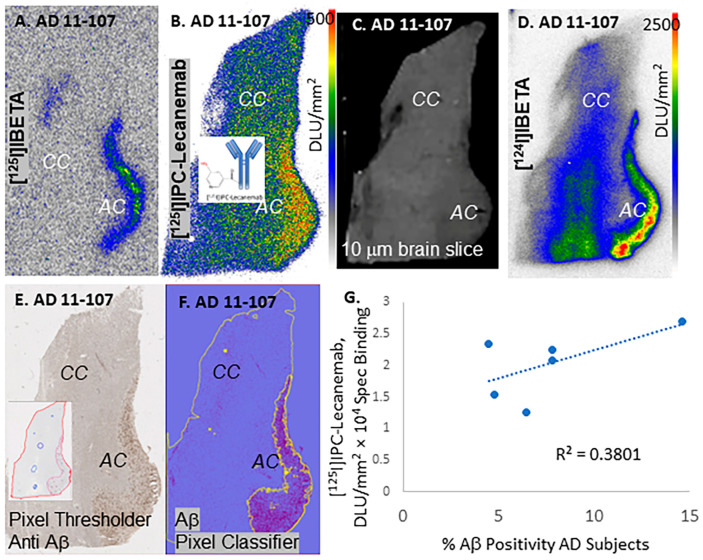
[^125^I]IPC-Lecanemab and [^125^I]IBETA binding in human postmortem AD. (**A**) [^125^I]IBETA binding to anterior cingulate (AC) of AD 11-107 (yellow-green-blue shows higher binding) with little binding to corpus callosum (CC) (grey-white shows nonspecific binding). (**B**) [^125^I]IPC-Lecanemab binding to AC of AD 11-107 (red-yellow shows higher binding) with nonspecific binding to corpus callosum (CC) (blue shows nonspecific binding). (**C**) Scan of brain slice showing AC and CC of AD 11-107. (**D**) [^124^I]IBETA binding to AC of AD 11-107 (red-yellow shows higher binding) with nonspecific binding to CC (blue). (**E**) Anti-Aβ IHC showing pixel thresholder in inset of AD 11-107. (**F**) Aβ pixel classifier image of AD 11-107 (purple shows Ab plaques identified by the pixel classifier). (**G**) Correlation plot of %Aβ plaque positivity and [^125^I]IPC-Lecanemab binding in six AD subjects.

The binding of the radioiodinated Aβ-plaque-imaging agents [^125^I]IBETA and [^124^I]IBETA was compared with that of [^125^I]IPC-Lecanemab in the AD subjects (Figure 4). A lower nonspecific binding in the corpus callosum was seen with [^125^I]IBETA compared to [^125^I]IPC-Lecanemab (Figure 4A,B). The average GM/WM ratio for [^125^I]IBETA was 10-fold higher (AC/CC = 20) across all the six AD subjects compared to [^125^I]IPC-Lecanemab. The binding of [^125^I]IBETA, [^124^I]IBETA and [^125^I]IPC-Lecanemab was consistent with anti-Aβ immunostaining (Figure 4E,F). Using QuPath, annotations were made on the Aβ plaques present in the anterior cingulate regions of each subject, as shown in Figure 4E,F. The percentage of Aβ positivity ranged from 4% to 15% across the six AD subjects.

There was a weak correlation between the binding of [^125^I]IPC-Lecanemab and %anti-Aβ positivity in the anterior cingulate. This may have been due to [^125^I]IPC-Lecanemab binding to Aβ protofibrils and Aβ fibrils in addition to Aβ plaques.

The [^124^I]IBETA autoradiographs were more intense due to the higher energy of iodine-124 photons compared to iodine-125 ones (Figure 4D). It may be expected that [^124^I]IPC-Lecanemab may provide higher AC/CC ratios due to its higher energy. This will have a positive impact on the image quality of [^124^I]IPC-Lecanemab for in vivo studies. It must be noted that buffer washing conditions post incubation with [^125^I]IPC-Lecanemab may improve the specific binding while reducing the nonspecific binding. None of the CN subjects exhibited any specific binding in the anterior cingulate, suggesting that the binding of [^125^I]IPC-Lecanemab was driven by the presence of Aβ in the AD subjects. All the AD subject brain samples used in the study were Braak stage V and VI. The degree to which these samples may contain Aβ protofibrils is uncertain.

## 4. Discussion

Immunotherapies targeting Aβ have gained immense attention during the last decade [32,33,34]. This has fostered research towards useful imaging agents for AD by using radiolabeled antibodies to target Aβ. Despite the large size of antibodies leading to low uptake, their high affinity for Aβ plaques with therapeutic implications makes imaging highly significant [32,33,34]. Several efforts have been made to increase their brain uptake [35,36,37,38]. These efforts alter the antibody targeting Aβ, such as conjugating to a transferrin receptor, to enable receptor-mediated transcytosis across the BBB for imaging mice models of AD [37,38]. Although the brain uptake of the antibodies is very low, preliminary efforts have been made to target a functionalized antibody that was preinjected in AD mice using the PET radioisotope copper-64 [39].

**Figure 5:**
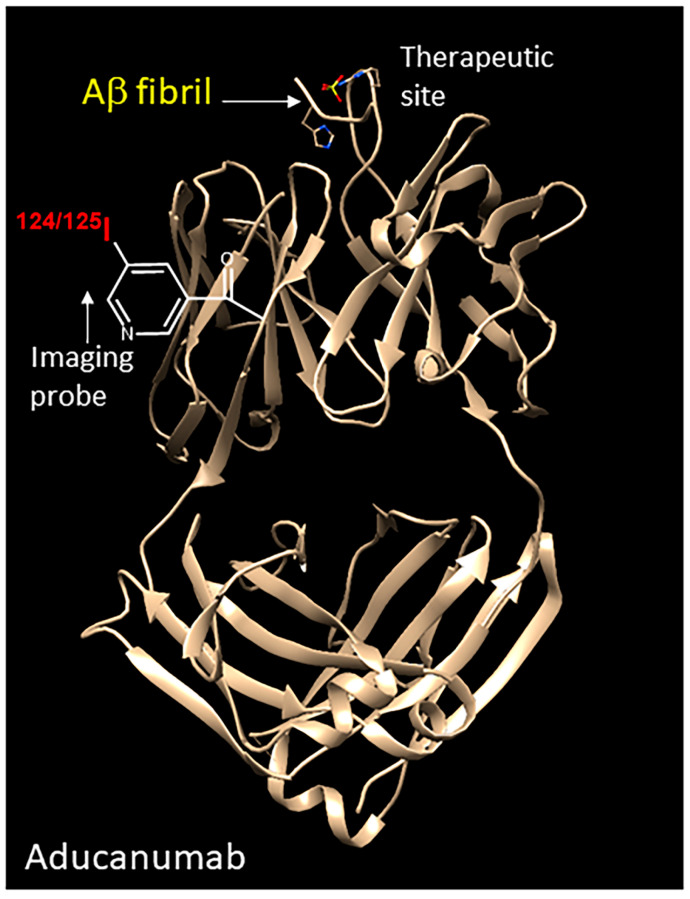
Aducanumab Aβ complex (PDB 6CO3 [32]). Structure of Aducanumab with the Ab fibril (yellow text) bound at the top. Similar to our results with [^125^I]IPC-Lecanemab, both Aducanumab and Lecanemab may be radiolabeled with iodine-124 (red text) to produce [^124^I]IPC-Aducanumab and [^124^I]IPC-Lecanemab, which can be used for PET imaging. Along with a focused ultrasound intervention, delivery of these therapeutic antibodies can be measured.

Lecanemab and Aducanumab have both shown promising response in the treatment of early-stage AD [4]. A comparison of Lecanemab with previously reported antibodies such as Aducanumab and Gantenerumab suggests a preferential affinity of Lecanemab for Aβ protofibrils. However, all the three antibodies have nanomolar affinities for small and large Aβ protofibrils and Aβ fibrils, while all of them have weak micromolar affinities for the Aβ monomer [3]. The structure of Aducanumab binding to aggregated Aβ has been reported, as shown in Figure 5 [32]. It has been suggested that Lecanemab has a similar interaction with the N-terminus of the Aβ peptide in a shallow pocket of the antibody surface. This unique binding of Aducanumab and Lecanemab compared to the less effective antibody Gantenerumab results in their greater effectiveness [3].

The radiolabeling of the murine versions of BAN2401 (Lecanemab), RmAb158 and RmAb158-scFv8D3, have been reported by adopting the bispecific imaging approach for increased brain uptake via transferrin-receptor-mediated transcytosis [30,31]. The antibodies were directly radiolabeled with iodine radioisotopes under oxidative conditions. Successful brain imaging in transgenic mice with the bispecific targeting approach was achieved. Our approach to obtain a radiolabeled Lecanemab involved the use of a bifunctional agent, SPC [24]. It was envisaged that the advantage of this agent would be to first radiolabel SPC with an iodine radioisotope under oxidative conditions. This radiolabeled SPC would then be coupled in an “amide” linkage to Lecanemab under milder conditions. This approach would provide a more stable radioiodine compared to labeling tyrosine residues, which is typically achieved in the direct labeling of antibodies with electrophilic iodine.

Our successful labeling of [^125^I]IPC-Lecanemab was possible under mild conditions. The ability of the radiolabeled [^125^I]IPC-Lecanemab to bind to the Aβ in AD brain slices is indicative of the preservation of the Aβ fibril binding site. Structural studies of Aducanumab have shown the binding of the Aβ protofibril at the top (Figure 5). A similar interaction has been suggested for Lecanemab [35,36]. Our results suggest that [^125^I]IPC-Lecanemab is a good imaging agent for Aβ fibrils and aggregates.

The comparison of [^125^I]IPC-Lecanemab with [^18^F]flotaza and [^125^I]IBETA showed a similarity in binding in the AD subjects. Because of the high nonspecific binding of [^125^I]IPC-Lecanemab, the GM-to-WM ratio was significantly lower for [^125^I]IPC-Lecanemab. However, it should be noted that these in vitro ratios cannot be directly translated to in vivo imaging ratios [32]. Nonetheless, a ratio of approximately 2 may be reasonable for human studies. Additionally, both [^18^F]flotaza and [^125^I]IBETA are known to bind to Aβ plaques with a high affinity, whereas [^125^I]IPC-Lecanemab binds to Aβ protofibrils with a high affinity. Since the AD subject brain samples in this study were in advanced stages of the disease, the amount of Aβ protofibrils may be small.

Iodine-124 is a long-half-life PET radioisotope that has been used for imaging various CNS targets [26]. We have successfully radiolabeled small molecules with iodine-124 for imaging Aβ plaques [15,28]. [^124^I]IBETA was used for PET/CT imaging of transgenic 5xFAD mice for Aβ plaques [15]. The in vivo binding of [^124^I]IBETA was correlated with in vitro binding and anti-Aβ immunostained brain slices. With our successful radiolabeling of Lecanemab with iodine-125, similar methods can be applied to prepare [^124^I]IPC-Lecanemab as well as [^124^I]IPC-Aducanumab (Figure 5).

**Figure 6:**
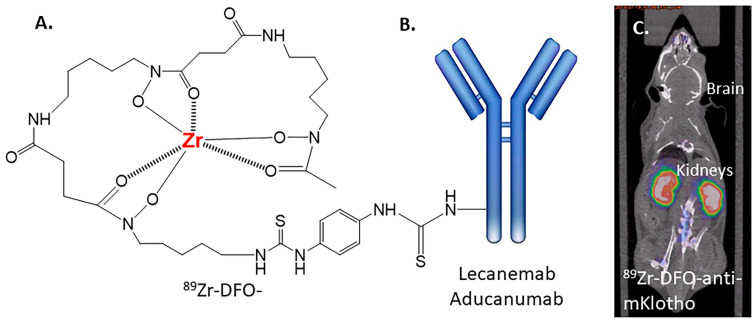
Zirconium-89-labeled antibodies. (**A**) Chemical structure of [^89^Zr]zirconium-deferoxamine DFO. (**B**) Potential structure [^89^Zr]DFO conjugated to Lecanemab or Aducanumab. (**C**) Mouse PET/CT imaging of labeled antibody [^89^Zr]DFO-anti-mKlotho, demonstrating strong radioactivity in the kidneys but lack of uptake in the brain [41].

A longer-half-life PET radioisotope, zirconium-89, is now being used along with a stable chelator, DFO, to label antibodies (Figure 6A). This methodology may be applied to Lecanemab and Aducanumab to provide a suitable radiolabeled Aβ antibody for PET imaging (Figure 6B). Our previous findings with ^89^Zr-DFO-anti-mKlotho, which was designed to specifically target the kidneys, show an absence of brain uptake indicative of a lack of BBB permeability (Figure 6C) [40]. This lack of brain uptake by the radiolabeled antibody is consistent with the observation of [^125^I]RmAb158, which is unable to permeate the BBB [32]. [^89^Zr]DFO-Adu-8D3 using the transferrin receptor to shuttle it across the BBB has therefore been used to successfully to image Aβ plaques in APP/PS1 mice brain [41].

Focused ultrasound is emerging as a viable alternative to temporarily make the BBB permeable for drug delivery, while mechanisms and safety investigations in preclinical and clinical use still continue to be evaluated [42,43,44]. Studies have been carried out using ultrasound and Aducanumab in APP23 transgenic AD mice [20]. The recent development of using focused ultrasound in AD patients to make the BBB permeable to therapeutic antibodies such as Aducanumab and Lecanemab appears to be promising [21]. Initial results on a limited number of subjects have shown reduced Aβ plaque load over several months of treatment with therapeutic doses of the antibodies [21]. The reduction in Aβ load post treatment (ultrasound and Aducanumab) was confirmed by small-molecule [^18^F]florbetaben imaging targeting the Aβ aggregates (Figure 1). Iodine-124-labeled Aducanumab and Lecanemab can be used in PET studies to measure the dosing of the therapeutic antibody to the brain regions during focused ultrasound treatment. This will provide a direct assessment of the binding and clearance of Aβ aggregates from the brain. Such an assessment may be useful in altering the course of antibody treatment as well as focused ultrasound.

## 5. Conclusions

A clinically useful therapeutic antibody, Lecanemab, has been successfully radiolabeled with iodine-125 using a bifunctional linker to provide [^125^I]IPC-Lecanemab. This new radiolabeled [^125^I]IPC-Lecanemab binds to Aβ aggregates in postmortem AD brains, suggesting the retention of its potential therapeutic value. In vivo imaging using iodine-124 (for PET) and iodine-123 (for SPECT) analogs of [^125^I]IPC-Lecanemab may be useful in dosing, monitoring and tailoring treatment strategies of AD patients using Lecanemab.

## Data Availability

The data that support the findings of this study are available from the corresponding author upon reasonable request.
